# miR-660-5p promotes breast cancer progression through down-regulating TET2 and activating PI3K/AKT/mTOR signaling

**DOI:** 10.1590/1414-431X20209740

**Published:** 2020-10-30

**Authors:** Bing Peng, Chao Li, Lili He, Mi Tian, Xin Li

**Affiliations:** 1 Department of Oncology, The Second People's Hospital of Jingmen, Jingmen, Hubei, China; 2 Department of General Surgery, The Second People's Hospital of Jingmen, Jingmen, Hubei, China; 3 Institute of Forensic Medicine, Jingmen Public Security Bureau, Jingmen, Hubei, China

**Keywords:** Breast cancer, miR-660-5p, TET2, PI3K/AKT/mTOR signaling

## Abstract

Breast cancer (BC) is a commonly diagnosed cancer in females. MicroRNA-660-5p (miR-660-5p) has been reported to be involved in the occurrence and development of BC. However, the regulatory network of miR-660-5p in BC has not been fully addressed. Quantitative real-time polymerase chain reaction (qRT-PCR) was performed to detect the enrichment of miR-660-5p and tet-eleven translocation 2 (TET2) in BC tissues and cells. Cell counting kit-8 (CCK8), flow cytometry, and transwell migration and invasion assays were used to measure cell proliferation, apoptosis, migration, and invasion. The target relationship between miR-660-5p and TET2 was confirmed by dual luciferase reporter assay. Protein expression was measured by western blot. The expression of miR-660-5p was elevated in BC, and high expression of miR-660-5p was closely related to lymph node metastasis, advanced TNM stage, and vascular invasion of BC tumors. miR-660-5p silencing inhibited cell proliferation and metastasis, but induced apoptosis of BC cells. TET2 was identified as a direct target of miR-660-5p, and the interference of TET2 partly reversed the suppressive effects of miR-660-5p silencing on the malignant potential of BC cells. miR-660-5p promoted BC progression partly through modulating TET2 and PI3K/AKT/mTOR signaling. miR-660-5p/TET2 axis might be a promising target for BC treatment.

## Introduction

Breast cancer (BC) is a malignant cancer with high risk in females. Tumor metastasis is a common cause of death of BC patients. The initiation and progression of BC are comprehensive outcomes of genetic and epigenetic factors. Although the therapeutic methods have been improved, the prognosis of patients with advanced BC remains poor. Therefore, research on pathogenesis and development of BC is crucial for BC treatment.

MicroRNAs (miRNAs) are a class of non-coding RNAs with 18-25 nucleotides in length that participate in the occurrence and progression of many cancers (1). They exert pivotal functions mainly by modulating gene expression at the post-transcriptional level, and miRNAs can bind to the 3′ untranslated region (3′ UTR) of the target messenger RNAs (mRNAs) and promote the degradation or translation repression of the corresponding mRNAs (2,3). Due to their abnormal expression and crucial functions in cancer progression, miRNAs serve as diagnostic indexes and prognosis biomarkers in a variety of cancers (4). Increasing evidence indicates that miR-660-5p might be a novel promising prognosis marker of BC. For instance, Krishnan et al. (5) reported that miR-660-5p abundance was closely correlated with proliferation, migration, and invasion of BC cells, and miR-660-5p might be a potential novel prognostic marker for BC treatment. According to Shen et al. (6), miR-660-5p is elevated in BC cells, and facilitates cell proliferation and metastasis but suppresses cell apoptosis through targeting transcription factor CP2 (TFCP2) in BC cells. Here, we identified a novel signal pathway by which miR-660-5p promoted the progression of BC.

miRNAs function through targeting and regulating the expression of mRNAs (2,3). Tet-eleven translocation (TET) family is a Fe^2+^ and α-ketoglutarate-dependent dioxygenase, including three members (TET1, TET2, and TET3) (7,8). TET2 acts as DNA demethylase to antagonize the DNMT-mediated gene methylation and gene expression inhibition. TET2 depletion up-regulates the methylation level and therefore silences many tumor suppressor genes. Therefore, TET2 plays a suppressive role in many kinds of cancers. Chen et al. (9) demonstrated that lysine demethylase KDM2A facilitates BC progression through promoting the methylation and silencing of tumor suppressor genes by inhibiting TET2. Zhu et al. (10) reported that TET2 suppressed BC development through regulating CASP4, and TET2 could enhance the expression of CASP4 to restrain tumorigenesis of BC. Here, the direct interaction between miR-660-5p and TET2 mRNA was identified in our study for the first time.

The phosphoinositide 3 kinase (PI3K)/AKT/protein kinase B (PKB)/mammalian target of rapamycin (mTOR) signaling is closely related to cell differentiation, proliferation, and apoptosis (11–14), and it is frequently activated in BC. Recent studies showed that the inhibition of PI3K/AKT/mTOR signaling is an effective method for the treatment of BC (15–17). In this study, the activity of PI3K/AKT/mTOR pathway was analyzed to explore if miR-660-5p/TET2 functions through regulating this signal pathway.

In this study, we firstly assessed the clinical significance of miR-660-5p in BC progression. Subsequently, the regulatory mechanism by which miR-660-5p modulated the proliferation, metastasis, and apoptosis of BC cells was explored.

## Material and Methods

### Tissue samples

Clinical research was authorized by the Ethics Committee of The Second People's Hospital of Jingmen, and all subjects signed informed consents before the radical excision operation. A total of 65 pairs of BC tissues and adjacent normal tissues were obtained from 65 BC patients who had never received chemotherapy, radiotherapy, or other treatments at The Second People's Hospital of Jingmen. Tissues were immediately stored in -80°C. The correlation between miR-660-5p expression and clinicopathological features in 65 patients with breast cancer is shown in Table 1.

**Table 1:** Correlation between miR-660-5p expression and clinicopathological features in 65 patients with breast cancer.

Parameters	n=65	miR-660-5p expression		P
		High (n=32)	Low (n=33)	
Age (years)				
≥50	40	22 (55.00%)	18 (45.00%)	1.385
<50	25	10 (40.00%)	15 (60.00%)	
Tumor size				
≥3 cm	42	23 (54.76%)	19 (45.24%)	0.228
<3 cm	23	9 (39.13%)	14 (60.87%)	
Lymph node metastasis				
Yes	39	25 (64.10%)	14 (35.90%)	0.003*
No	26	7 (26.92%)	19 (73.08%)	
Clinical TNM stage				
I-II	35	12 (34.29%)	23 (65.71%)	0.009*
III-IV	30	20 (66.67%)	10 (33.33%)	
Differentiation				
Well/moderate	28	10 (35.71%)	18 (64.29%)	0.058
Poor	37	22 (59.46%)	15 (40.54%)	
Vascular invasion				
Yes	29	19 (65.52%)	10 (34.48%)	0.018*
No	36	13 (36.11%)	23 (63.89%)	

Data are reported as number and percentage. *P<0.05 (chi-squared test).

### Cell culture

Human breast epithelial cell line MCF-10A and human BC cell lines MCF-7 and MDA-MB-231 were obtained from the American Type Culture Collection (ATCC, USA). MCF-10A cell line was maintained in Dulbecco's modified Eagle medium (DMEM)/Nutrient mixture F-12 Ham medium containing 100 ng/mL cholera toxin, 20 ng/mL EGF, 10 µg/mL insulin, 500 ng/mL hydrocortisone, and 5% horse serum (Sigma, USA). DMEM supplemented with 10% fetal bovine serum (Gibco, USA) was used for the cultivation of BC cell lines. All cell lines were cultured at 37°C in a 5% CO_2_ incubator.

### Cell transfection

TET2 specific small interfering RNA (si-TET2), scrambled (negative control) siRNA (si-NC), TET2 overexpression plasmid (oeTET2; Gene ID: 54790; NCBI Reference Sequence: NM_001127208.3), and empty vector (vector) were purchased from GenePharma (China). miR-660-5p mimics, mimics NC, miR-660-5p inhibitor, and inhibitor NC were obtained from Ribobio (China). Transfection was carried out with Lipofectamine 3000 (Invitrogen, USA).

### Quantitative real-time polymerase chain reaction (qRT-PCR)

RNA sample was extracted using TRIzol solution (Invitrogen). The reverse transcription was carried out with M-MLV reverse transcriptase kit (for TET2; Invitrogen) and All-in-One^TM^ miRNA First stand cDNA Synthesis kit (for miR-660-5p; GeneCopoeia, USA). Special primers purchased from GeneCopoeia are listed below. SYBR Premix Taq^TM^ II kit (Takara, China) was used in PCR reaction. The abundance of miR-660-5p (U6 acted as the control) and TET2 (glyceraldehyde-3-phosphate dehydrogenase (GAPDH) used as the control) was analyzed by the 2^-ΔΔCt^ method (18). miR-660-5p (forward, 5′-TACCCATTGCATATCGGAGTTG-3′; reverse, universal primer), TET2 (forward, 5′-GGACTGAGCTGCTGAATTCAACT-3′; reverse, 5′-CCTCAACATGGTTGGTTCTATCC-3′), U6 (forward, 5′-CTCGCTTCGGCAGCACA-3′; reverse, 5′-AACGCTTCACGAATTTGCGT-3′), and GAPDH (forward, 5′-CTGGGCTACACTGAGCACC-3′; reverse, 5′-AAGTGG TCGTTGAGGGCAATG-3′).

### Cell proliferation detection by cell counting kit-8 (CCK8) assay

Cell counting kit-8 (Sigma) was used to analyze the proliferation capacity of BC cells. BC cells (5000 cells/well) were seeded into 96-well plates to settle down overnight. After transfection for an interval of 24 h, 15 µL CCK8 solution was incubated with the viable BC cells for 2 h at room temperature. The absorbance was analyzed using microplate reader (Molecular Devices, USA) at 450 nm.

### Cell apoptosis analysis

BC cells (3×10^5^ cells/well) were plated into 6-well plates to settle down overnight. After specific transfection for 72 h, BC cells were collected using cold phosphate buffer saline (PBS) buffer twice. BC cells were simultaneously stained with 5 µL Annexin V combined FITC and PI (Solarbio, China) to mark the phosphatidylserine (PS) and DNA content. The early (FITC^+^/PI^-^) and late apoptotic cells (FITC^+^/PI^+^) were distinguished from normal and necrotic cells by the flow cytometer (Beckman Coulter, USA).

### Transwell migration and invasion assay

Transwell chambers with 8-µM pore filters (Corning Inc., USA) were used in the transwell migration and invasion assay to analyze the metastasis ability of BC cells.

In the transwell migration assay, after transfection for 24 h, BC cells (1×10^4^ cells/100 µL serum-free medium) were seeded into the uncoated upper chambers. Fetal bovine serum (FBS, 10%) in medium was added to fill the lower chambers to act as the chemoattractant. After 24-h incubation, un-migrated BC cells in the upper surface were scraped using a cotton swab, and migrated BC cells were fixed using 4% paraformaldehyde (Sangon Biotech, China) and dyed using crystal violet (Sangon Biotech). The number of migrated BC cells in five random fields was counted and averaged.

In the transwell invasion assay, 40 µL BD Matrigel matrix (BD Biosciences, USA) diluted in cold serum-free medium of 1:8 was used to pre-coat the upper chambers to simulate the extracellular matrix before seeding BC cells (7×10^4^ cells/100 µL serum-free medium; after transfection for 24 h), and the remaining procedures were similar with transwell migration assay.

### Dual-luciferase reporter assay

The spatial interaction between miR-660-5p and TET2 was predicted by the TargetScan database (http://www.targetscan.org). Dual-luciferase reporter assay was conducted to verify the target interaction between miR-660-5p and TET2.

The partial sequence in the 3′ UTR of TET2 (Gene ID: 54790; 3′UTR 1502 site to 1914 site; 413 bp), containing the wild-type (WT) binding sites or mutant (MUT) binding sites with miR-660-5p, was cloned into the pmirGLO vector (Promega, USA) to generate TET2 WT or TET2 MUT. BC cells in 24-well plates were co-transfected with 10 nM miR-660-5p mimics or mimics NC and 40 ng TET2 WT or TET2 MUT. After 48-h transfection, the luciferase activities were measured using the dual-luciferase reporter assay system kit (Promega) with the luminometer (Plate Chameleon V, Finland). Renilla fluorescence intensity was used as the control.

### Western blot assay

BC cells were lysed with RIPA lysis solution (Beyotime, China). Quantified protein samples were separated via sodium dodecyl sulfate polyacrylamide gel electrophoresis (SDS-PAGE). Subsequently, the proteins were blotted onto the polyvinylidene fluoride (PVDF) membrane (Millipore, USA). After blocking for 1 h, the PVDF membrane was probed with the following primary antibodies against TET2 (ab94580, Abcam, USA), GAPDH (ab37168, Abcam), p-AKT (T308; ab38449, Abcam), AKT (ab8805, Abcam), p-mTOR (ab109268, Abcam), or mTOR (ab2732, Abcam), and HRP combined secondary antibody (ab6721, Abcam) was used to probe the membrane for 2 h. The intensity of protein bands was analyzed using the enhanced chemiluminescence (ECL) system.

### Murine xenograft assay

Nude mouse xenograft experiments were approved by the Animal Research Committee of The Second People's Hospital of Jingmen. Tumor xenograft model was built using MCF-7 cells transfected with miR-660-5p inhibitor or inhibitor NC or un-transfected MCF-7 cells. The above MCF-7 cells (2×10^6^ cells/200 µL PBS) were subcutaneously injected into the right side of the back in nude mice (n=5), and the tumor volume was measured every week. Four weeks after injection, the nude mice were sacrificed, and the weight of tumors was recorded. Tumor tissues were used to detect the levels of miR-660-5p, TET2, and the activation of PI3K/AKT/mTOR signaling.

### Statistical analysis

All experiments were repeated three times, and there were at least three technical replicates in each experiment. All statistical data are reported as means±SD. Student's *t*-test and one-way analysis of variance (ANOVA) were used to compare two groups or multiple groups. P<0.05 was defined as statistically significant.

## Results

### miR-660-5p was enhanced in BC

**Figure 1:**
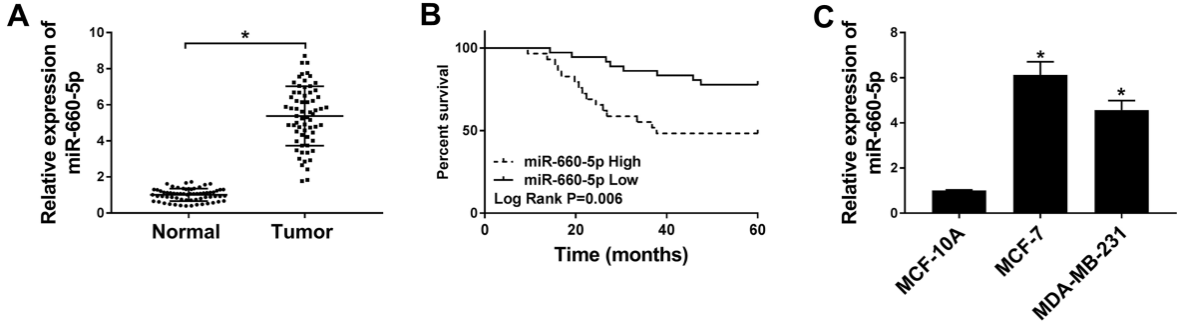
A, The level of miR-660-5p was measured in breast cancer (BC) tissues (n=65) and corresponding normal tissues (n=65) by qRT-PCR. **B**, The survival rate was analyzed in miR-660-5p high and low expression BC patients using Kaplan-Meier analysis and log-rank test. **C**, The abundance of miR-660-5p was detected in normal human breast epithelial cell MCF-10A (n=3) and BC cells (n=3) by qRT-PCR. Data are reported as means±SD. *P<0.05 (ANOVA).

The expression of miR-660-5p in BC tissues and cells was measured by qRT-PCR. As shown in Figure 1A and C, miR-660-5p level was significantly increased in BC tissues and cells compared with that in adjacent normal tissues and human breast epithelial cells MCF-10A. As shown in Figure 1B, high miR-660-5p expression was associated with short survival time of BC patients. These data showed that miR-660-5p might an exert important role in BC progression.

### miR-660-5p depletion suppressed proliferation and metastasis of BC cells

**Figure 2:**
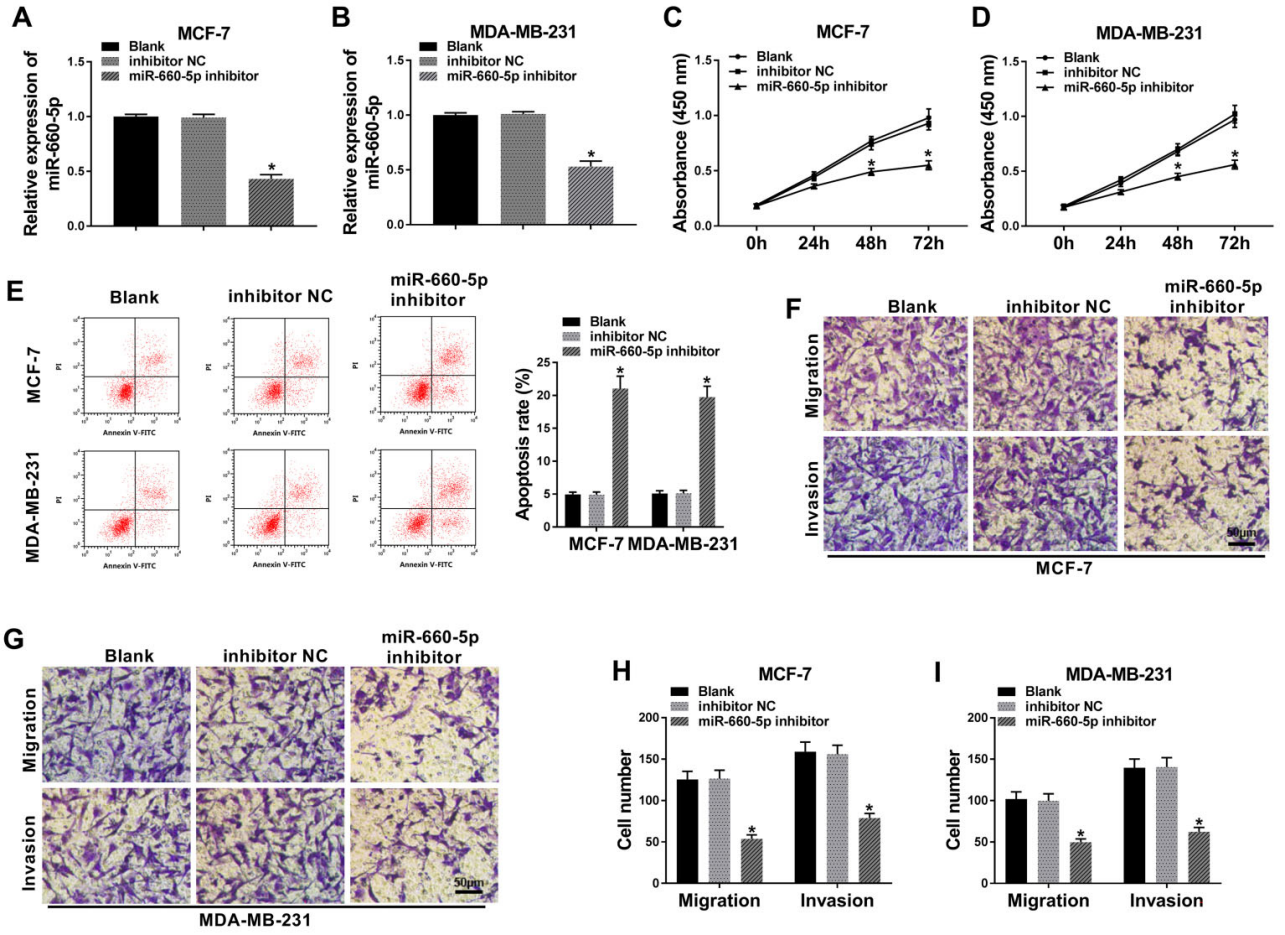
Aand **B**, The knockdown efficiency of miR-660-5p inhibitor was measured in MCF-7 and MDA-MB-231 cells (n=3) by qRT-PCR after transfection for 24 h. **C**-**I**, MCF-7 and MDA-MB-231 cells were transfected with inhibitor negative control (NC) or miR-660-5p inhibitor. **C** and **D**, Cell proliferation was examined by CCK8 assay. Breast cancer (BC) cells were seeded into 96-well plates (n=6), and cell proliferation curve was generated through measuring the absorbance at 0, 24, 48, or 72 h after transfection. **E**, BC cells were seeded into 6-well plates (n=3), and flow cytometry was performed to assess apoptosis after transfection for 72 h. **F**-**I**, Cell migration and invasion were measured in BC cells (n=3) by transwell migration and invasion assays after transfection for 24 h. Data are reported as means±SD. *P<0.05 (ANOVA).

To explore the biological role of miR-660-5p in BC cells, we performed loss-of-function experiments. Transfection with miR-660-5p inhibitor significantly reduced the enrichment of miR-660-5p compared with the blank and inhibitor NC groups in MCF-7 and MDA-MB-231 cells (Figure 2A and B). As indicated in Figure 2C and D, cell proliferation was prominently suppressed in MCF-7 and MDA-MB-231 cells transfected with miR-660-5p inhibitor compared with that in the control group. In addition, the depletion of miR-660-5p accelerated cell apoptosis (Figure 2E). As shown in Figure 2F-I, transwell migration and invasion assays demonstrated that the inhibition of miR-660-5p suppressed cell metastasis. Taken together, miR-660-5p facilitated proliferation and metastasis while it impeded cell apoptosis of BC cells. Therefore, miR-660-5p played an oncogenic role in BC cells.

### TET2 was a direct target of miR-660-5p

**Figure 3:**
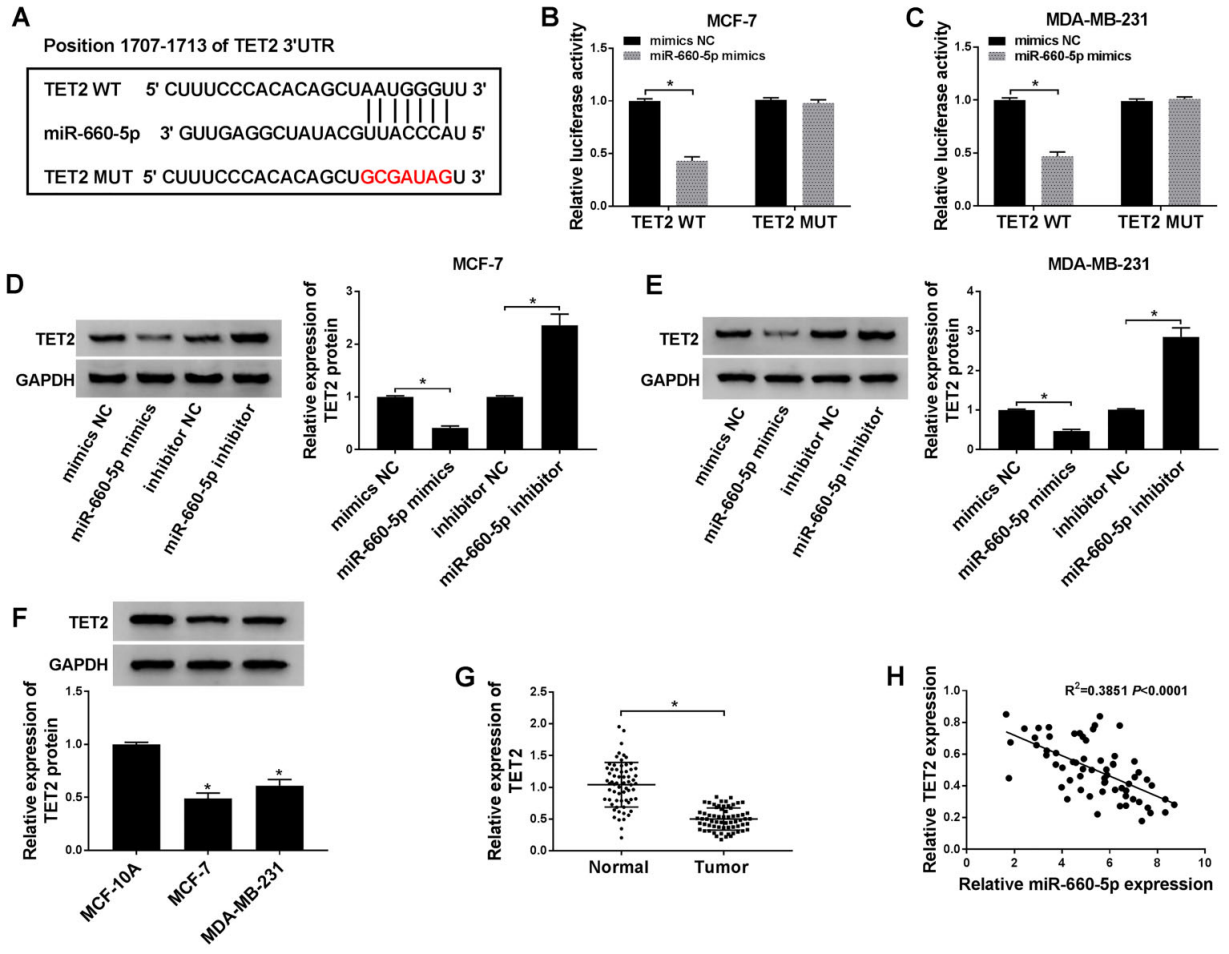
A, The binding sites between miR-660-5p and TET2 were predicted by TargetScan online software. **B** and **C**, Luciferase activity was determined in MCF-7 and MDA-MB-231 cells (n=3) co-transfected with TET2 wild type (WT) or TET2 mutant (MUT) and mimics negative control (NC) or miR-660-5p mimics for 48 h. **D** and **E**, The expression level of TET2 was measured in breast cancer (BC) cells (n=3) transfected with mimics NC, miR-660-5p mimics, inhibitor NC, or miR-660-5p inhibitor for 24 h by western blot. **F**, The enrichment of TET2 was detected in BC cells (n=3) and normal human breast epithelial cells MCF-10A (n=3) by western blot. **G**, The expression of TET2 was measured in BC (n=65) and normal tissues (n=65) by qRT-PCR. **H**, The relationship between the expression of miR-660-5p and TET2 was analyzed in BC tissues. Data are reported as means±SD. *P<0.05 (Student's *t*-test and ANOVA).

As indicated in Figure 3A, the 3′ UTR of TET2 mRNA was found to bind to the ‘seed' sites of miR-660-5p, and this relationship was then confirmed by dual-luciferase reporter assay. The luciferase activity was significantly decreased in MCF-7 and MDA-MB-231 cells co-transfected with miR-660-5p mimics and TET2 WT plasmid, whereas it remained unchanged in cells co-transfected with miR-660-5p mimics and TET2 MUT, demonstrating that miR-660-5p directly bound to TET2 in the two cell lines (Figure 3B and C). To further evaluate the regulatory mechanism between miR-660-5p and TET2 in MCF-7 and MDA-MB-231 cells, western blot was applied to measure the expression of TET2 after transfection with miR-660-5p mimics or miR-660-5p inhibitor. The expression of TET2 was significantly decreased by the overexpression of miR-660-5p. In contrast, miR-660-5p depletion up-regulated the level of TET2 in MCF-7 and MDA-MB-231 cells (Figure 3D and E). Accordingly, TET2, as a functional target of miR-660-5p, was inversely modulated by miR-660-5p in BC cells. We assessed the abundance of TET2 in BC cells and tissues using western blot and qRT-PCR. The level of TET2 was lower in BC cells and tissues compared to that in normal human breast epithelial cells MCF-10A and adjacent normal tissues (Figure 3F and G). The enrichment of TET2 was negatively correlated with the level of miR-660-5p in BC tissues (Figure 3H). Therefore, TET2 was negatively regulated by miR-660-5p and might play a suppressive role in BC.

### TET2 overexpression suppressed growth and metastasis and promoted apoptosis of BC cells

**Figure 4:**
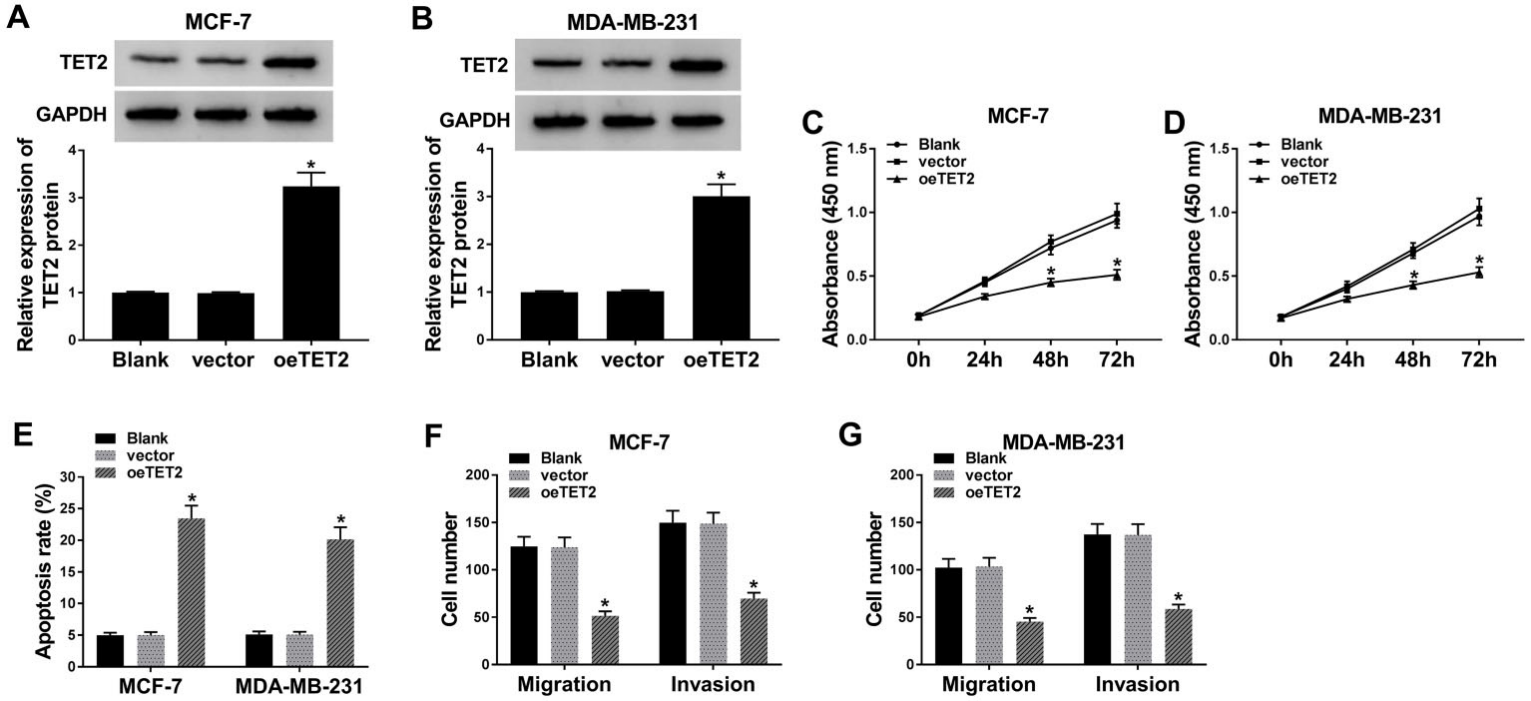
MCF-7 and MDA-MB-231 cells were transfected with vector or TET2 overexpression plasmid (oeTET2). **A** and **B**, The expression of TET2 was detected in MCF-7 and MDA-MB-231 cells (n=3) by qRT-PCR after transfection for 24 h. **C** and **D**, CCK8 assay was conducted to measure the proliferation of breast cancer (BC) cells (n=6). **E**, Cell apoptosis was evaluated in the two cell lines (n=3) by flow cytometry after transfection for 72 h. **F** and **G**, Cell migration and invasion were assessed by transwell migration and invasion assays after transfection for 24 h (n=3). Data are reported as means±SD. *P<0.05 (ANOVA).

TET2 was notably elevated with the transfection of TET2 overexpression vector (oeTET2) (Figure 4A and B). As shown in Figure 4C and D, the overexpression of TET2 decreased proliferation of MCF-7 and MDA-MB-231 cells. Cell apoptosis was significantly increased in BC cells transfected with oeTET2 (Figure 4E). As shown in Figure 4F and G, the accumulation of TET2 impeded cell migration and metastasis in MCF-7 and MDA-MB-231 cells. Accordingly, TET2 played a tumor suppressor role to promote apoptosis and restrain proliferation, migration, and invasion of BC cells.

### TET2 interference eliminated the inhibition effects of miR-660-5p depletion on proliferation and metastasis of BC cells

**Figure 5:**
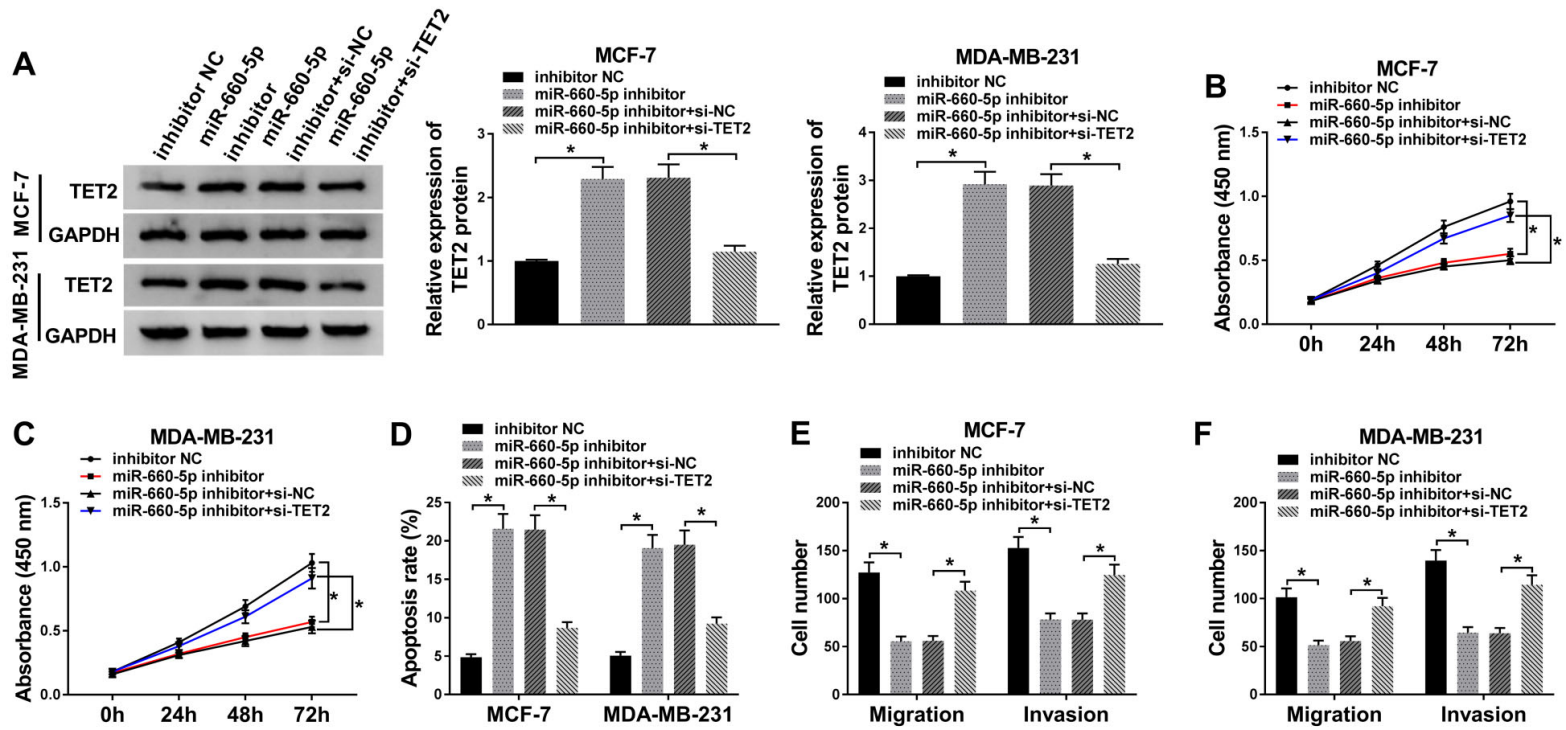
MCF-7 and MDA-MB-231 cells were transfected with inhibitor negative control (NC), miR-660-5p inhibitor, or miR-660-5p inhibitor plus si-NC or si-TET2. **A**, The expression of TET2 was determined in breast cancer (BC) cells by western blot after transfection for 24 h (n=3). **B** and **C**, CCK8 assay was carried out to detect cell proliferation in BC cells (n=6). **D**, Cell apoptosis was assessed in BC cells by flow cytometry after transfection for 72 h (n=3). **E** and **F**, Cell metastasis was evaluated in BC cells by transwell migration and invasion assays after transfection for 24 h (n=3). Data are reported as means±SD. *P<0.05 (ANOVA).

The level of TET2 was up-regulated by the addition of miR-660-5p inhibitor and decreased by the co-transfection of si-TET2 and miR-660-5p inhibitor in the two cell lines (Figure 5A). The interference of TET2 partly counteracted the inhibition impact of miR-660-5p depletion on cell proliferation (Figure 5B and C). In addition, the transfection of si-TET2 partly reversed the promoting effect of miR-660-5p depletion on cell apoptosis in the two cell lines (Figure 5D). Transwell migration and invasion assays suggested that the intervention of TET2 partly abolished the inhibiting impact of miR-660-5p inhibition on cell metastasis in MCF-7 and MDA-MB-231 cells (Figure 5E and F). Furthermore, the abundance of N-cadherin was decreased in BC cells with the interference of miR-660-5p, and the addition of si-TET2 partly recovered the protein level of N-cadherin (Figure S1A and B). The expression of E-cadherin exhibited an opposite trend to N-cadherin (Figure S1A and B), suggesting that miR-660-5p accelerated the metastasis of BC cells at least partly through targeting TET2. Overall, miR-660-5p facilitated the progression of BC at least through targeting TET2.

### TET2 knockdown abrogated the suppressive impact of miR-660-5p depletion on PI3K/AKT/mTOR signaling

**Figure 6:**
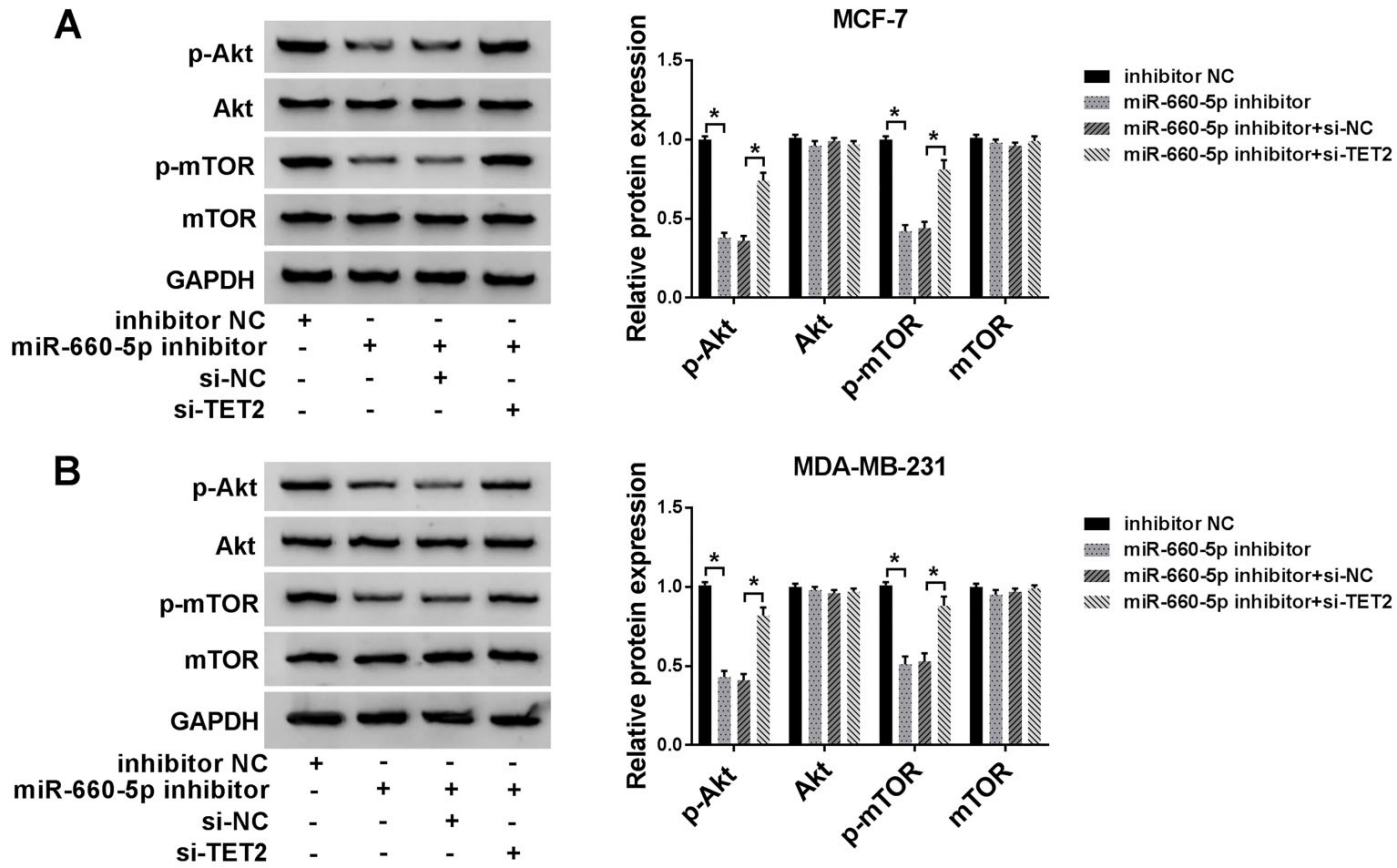
Phosphorylation of AKT, AKT, phosphorylation of mTOR, and mTOR were detected by western blot in MCF-7 (**A**) and MDA-MB-231 (**B**) cells transfected with inhibitor negative control (NC), miR-660-5p inhibitor, or miR-660-5p inhibitor plus si-NC or si-TET2 for 24 h (n=3). Data are reported as means±SD. *P<0.05 (ANOVA).

As expected, the depletion of miR-660-5p down-regulated the phosphorylation of AKT and mTOR, and the addition of si-TET2 restored the phosphorylation levels of AKT and mTOR (Figure 6A and B). According to the above results, it was shown that miR-660-5p facilitated proliferation and metastasis of BC cells by down-regulating TET2 and activating PI3K/AKT/mTOR signaling.

### miR-660-5p depletion inhibited tumor growth *in vivo*

**Figure 7:**
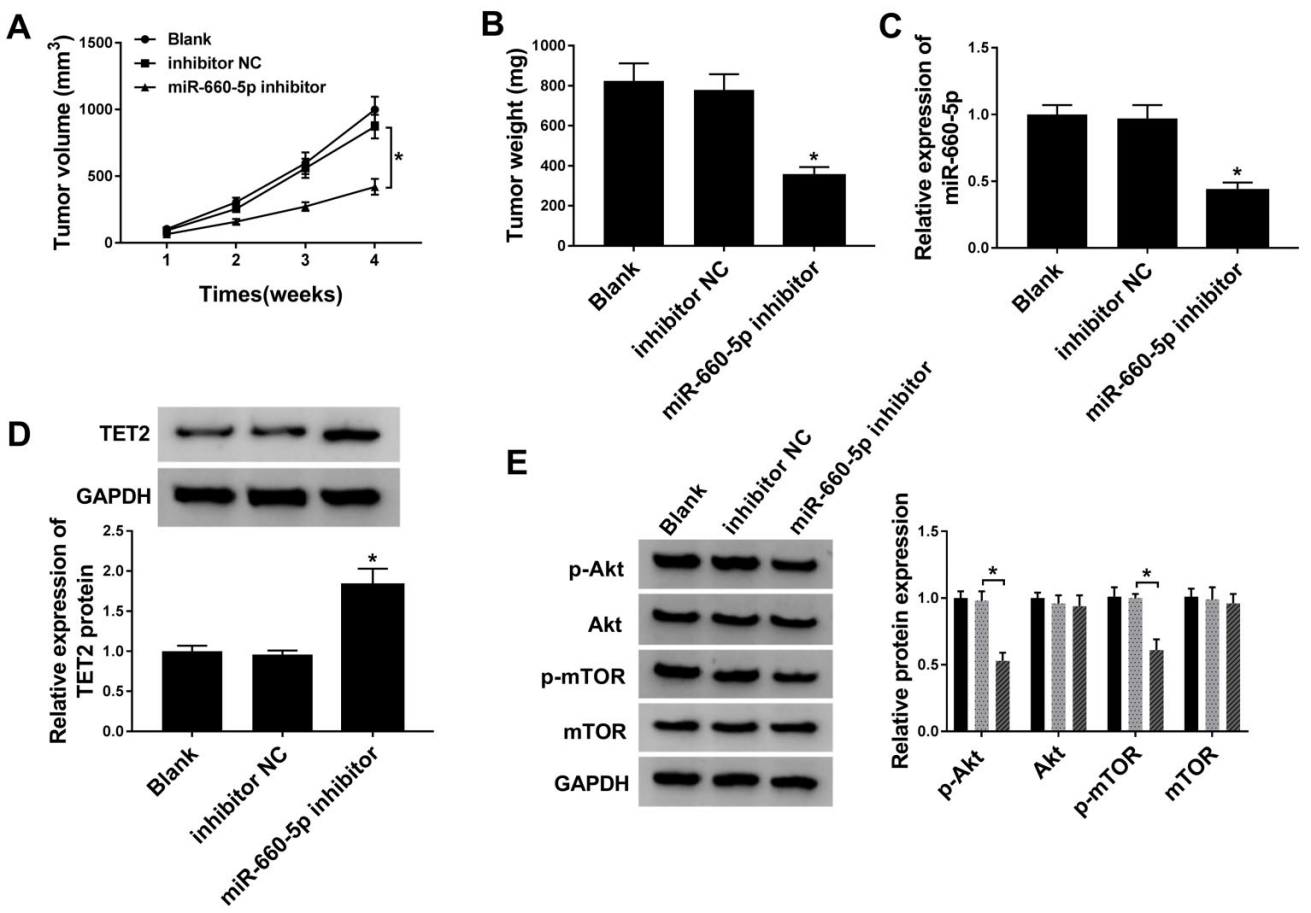
MCF-7 cells transfected with miR-660-5p inhibitor or inhibitor negative control (NC) and un-transfected MCF-7 cells were subcutaneously injected into the right side of the back in the nude murine (n=5). **A**, Tumor volume was recorded once a week. **B**, Murine xenograft tumor was weighed four weeks after inoculation. **C**, The expression of miR-660-5p was analyzed in resected tumor tissues by qRT-PCR. **D**, The protein level of TET2 was determined in resected tumor tissues by western blot. **E**, The enrichment of p-AKT, AKT, p-mTOR, and mTOR was examined by western blot. Data are reported as means±SD. *P<0.05 (ANOVA).

Tumor volume and weight were lower in the miR-660-5p inhibitor group than in the inhibitor NC and blank groups (Figure 7A and B). As shown in Figure 7C, the expression of miR-660-5p was down-regulated in the miR-660-5p inhibitor group compared with that in the inhibitor NC and blank groups. The abundance of TET2 was negatively connected with the level of miR-660-5p in tumor tissues (Figure 7D). Furthermore, the phosphorylation of AKT and mTOR was significantly decreased in tumor tissues transfected with miR-660-5p inhibitor than in the inhibitor NC and blank groups (Figure 7E). Therefore, these data suggested that miR-660-5p depletion suppressed BC tumor growth through targeting TET2 and regulating PI3K/AKT/mTOR signaling *in vivo*.

## Discussion

In the present study, we found that miR-660-5p could bind to TET2, and miR-660-5p facilitated proliferation and metastasis of BC cells through down-regulating TET2 and activating PI3K/AKT/mTOR signaling.

Accumulating articles have pointed out that dysregulated miRNAs are associated with the pathogenesis and progression of cancers (19,20). miRNAs function as oncogenes or tumor suppressors to restrain the expression of downstream genes related to proliferation, metastasis, apoptosis, and differentiation, thus regulating cellular biological processes (21). The expression of miR-660-5p is elevated in many types of cancer. For instance, Zhang et al. (22) reported that miR-660-5p is overexpressed in osteosarcoma, and it promotes proliferation and invasion of osteosarcoma cells by down-regulating its target forkhead box O1 (FOXO1). Qi et al. (23) found that the abundance of miR-660-5p is enhanced in the plasma and exosomes of non-small cell lung cancer (NSCLC) patients, and miR-660-5p might promote proliferation and metastasis of NSCLC cells by reducing the abundance of KLF9. Shen et al. (6) claimed that miR-660-5p inhibition restrains the progression and motility of BC cells. qRT-PCR results revealed that the enrichment of miR-660-5p was higher in BC tissues compared with that in adjacent normal tissues. The increased expression of miR-660-5p was closely related to lymph node metastasis, advanced TNM stage, and vascular invasion (Table 1) together with poor prognosis. The interference of miR-660-5p in MCF-7 and MDA-MB-231 suppressed cell growth and metastasis and promoted apoptosis, and the oncogenic role of miR-660-5p in BC was consistent with the previous research (6). However, the mechanism by which miR-660-5p mediates the proliferation, metastasis and apoptosis of BC cells remains unclear. Shen et al. (6) found that transcription factor CP2 (TFCP2) is a target of miR-660-5p, and miR-660-5p accelerated the progression of BC through targeting TFCP2. In the current study, the other target of miR-660-5p named TET2 was predicted by TargetScan online software, and dual-luciferase reporter assay verified the relationship between miR-660-5p and TET2. The level of TET2 was found to be negatively regulated by miR-660-5p in MCF-7 and MDA-MB-231 cells.

TET2 is a member of the TET dioxygenases family, and it is also known as DNA demethylase. TET2 mutation or depletion promoted tumorigenesis by enhancing the methylation level of tumor suppressor genes and down-regulating their expression levels. The report of Chiba showed that the mutation of TET2 is closely related to the initiation of hematologic malignancies (24). Nickerson et al. (25) reported that TET2 interference facilitated proliferation and migration of prostate cancer (PCa) cells. Apart from these, Chen et al. (9) and Zhu et al. (10) also reported that the depletion of TET2 promoted the development of BC. Consistent with the above results, we found that the accumulation of TET2 inhibited cell growth and metastasis but induced cell apoptosis in BC cells. The knockdown of TET2 partly counteracted the suppressive effects of miR-660-5p depletion on the malignant behaviors of BC cells.

How does miR-660-5p/TET2 axis work on proliferation, motility, and apoptosis of BC cells? PI3K/AKT/mTOR signal pathway is involved in cell survival, proliferation, migration, and invasion. The aberrant activation or mutation of PI3K/AKT/mTOR signal pathway was frequently detected in BC (26 [Bibr B27] [Bibr B28] [Bibr B29] [Bibr B30]). Many efforts were conducted to explore the inhibitors of PI3K/AKT/mTOR signal pathway in BC to improve the prognosis of patients (15,). We found that TET2 interference alleviated the down-regulation of p-AKT and p-mTOR caused by miR-660-5p inhibition.

Murine xenograft model validated that miR-660-5p interference suppressed the growth of BC tumors *in vivo*. However, the mechanism by which DNA demethylase TET2 modulates the activation of PI3K/AKT/mTOR pathway remains elusive. More detail about the regulation mechanism between TET2 and PI3K/AKT/mTOR signaling should be studied in the future. Furthermore, the PI3K/AKT signaling inhibitor was needed to verify the functional association between miR-660-5p/TET2 axis and PI3K/AKT signaling in the future.

In summary, this study elucidated the oncogenic effect of miR-660-5p in BC. miR-660-5p promoted proliferation and motility but suppressed cell apoptosis of BC cells through activating PI3K/AKT/mTOR signaling by down-regulating TET2. miR-660-5p/TET2 might be an underlying therapeutic target for BC treatment.

## Supplementary Material

Click here to view [pdf].
